# Comment on Hurley, J.C. Towards Clinical Application of Anti-endotoxin Antibodies; A Re-Appraisal of the Disconnect. *Toxins* 2013, *5*, 2589-2620

**DOI:** 10.3390/toxins6041362

**Published:** 2014-04-11

**Authors:** John G. Brock-Utne

**Affiliations:** Department of Anesthesiology, Perioperative and Pain Medicine, Stanford University School of Medicine Stanford, CA 94305, USA; E-Mail: brockutn@stanford.edu; Tel.: +1-650-498-4901; Fax: +1-650-725-8544

I have read with interest James C. Hurley’s very good review [1]. I totally agree that a reappraisal of the use of anti-endotoxin antibodies in Gram negative infections is warranted. In a study [2] we showed the possible association between endotoxin (LPS) and morbidity and mortality in septic shock. This was a study in healthy primates (vervet monkeys). We found, when these anesthetized primates received an LD100 iv infusion of *Echerichia coli* (*E. coli*) over one hour, both *E. coli* and endotoxin concentration significantly increased during the *E. coli* infusion. The anti-endotoxin (anti-LPS) on the other hand decreased significantly. Interestingly, when the animals succumbed, their LPS concentration was still raised, but there were no viable *E. coli.* There was also only a small amount of anti-LPS present. Hence, endotoxin concentration rather than circulating *E. coli* bacteria may be an important pathogen responsible for the high mortality experienced during *E. coli* shock. This is in agreement with Spink *et al.* [3] and now Hurley [1] who suggested that endotoxin which forms an integral part of the outer cellular membrane of gram negative bacteria (GNB) participates in the genesis of shock.

In our review [4] and some of the other papers we published in this field [5,6,7,8,9], we refer to successful preliminary studies using anti-lipopolysaccharide IgG (anti-LPS). The anti-LPS both present prior to the insult or given after the insult, would seem to inactivate plasma endotoxins and combat Gram-negative bacteria in sepsis. Thereby, as Hurley [1] suggests, may form part of a possible new form of therapy. 

The question that needs to be addressed is: how best to accomplice this? What part of the endotoxin should be attacked, the *O*-specific chain or the smaller Lipid-A, or even, if possible, both? 
